# Parallelism of DOG1 expression with recurrence risk in gastrointestinal stromal tumors bearing *KIT* or *PDGFRA* mutations

**DOI:** 10.1186/s12885-016-2111-x

**Published:** 2016-02-11

**Authors:** Francesca Maria Rizzo, Raffaele Palmirotta, Andrea Marzullo, Nicoletta Resta, Mauro Cives, Marco Tucci, Franco Silvestris

**Affiliations:** Department of Biomedical Sciences and Human Oncology, University of Bari “A. Moro”, Piazza Giulio Cesare, 11-70124 Bari, Italy; University San Raffaele Rome, Interinstitutional Multidisciplinary BioBank (BioBIM), IRCCS San Raffaele Pisana, Rome, Italy; Department of Pathology, University of Bari “A. Moro”, Bari, Italy; Division of Medical Genetics, Department of Biomedical Sciences and Human Oncology, University of Bari “A. Moro”, Bari, Italy

**Keywords:** Gastrointestinal stromal tumors, DOG1, Size, Mutation, Prognostic value, Risk

## Abstract

**Background:**

Gastrointestinal stromal tumors (GISTs) are characterized by mutations of *KIT* (v-kit Hardy-Zuckerman 4 feline sarcoma viral oncogene homolog) or *PDGFRA* (platelet-derived growth factor receptor α) that may be efficiently targeted by tyrosine kinase inhibitors (TKI). Notwithstanding the early responsiveness to TKI, the majority of GISTs progress, imposing the need for alternative therapeutic strategies. DOG1 (discovered on GIST-1) shows a higher sensitivity as a diagnostic marker than KIT, however its prognostic role has been little investigated.

**Methods:**

We evaluated DOG1 expression by immunohistochemistry (IHC) in 59 patients with GISTs, and correlated its levels with clinical and pathological features as well as mutational status. Kaplan-Meier analysis was also applied to assess correlations of the staining score with patient recurrence-free survival (RFS).

**Results:**

DOG1 was expressed in 66 % of CD117^+^ GISTs and highly associated with tumor size and the rate of wild-type tumors. Kaplan-Meier survival analysis showed that a strong DOG1 expression demonstrated by IHC correlated with a worse 2-year RFS rate, suggesting its potential ability to predict GISTs with poor prognosis.

**Conclusions:**

These findings suggest a prognostic role for DOG1, as well as its potential for inclusion in the criteria for risk stratification.

**Electronic supplementary material:**

The online version of this article (doi:10.1186/s12885-016-2111-x) contains supplementary material, which is available to authorized users.

## Background

Gastrointestinal stromal tumors (GISTs) develop within the digestive tract and harbor functional mutations of *KIT* (v-kit Hardy-Zuckerman 4 feline sarcoma viral oncogene homolog) and *PDGFRA* (platelet-derived growth factor receptor-α) that primarily drive the tumor growth and progression [1, 2]. *KIT* and *PDGFRA* genes are located on the chromosome 4q12 and encode transmembrane glycoproteins belonging to the type III receptor tyrosine kinase family. They are normally activated by their ligands, namely stem cell factor and PDGF respectively, which bind the receptor extracellular domain leading to the dimerization of receptors and phosphorylation of tyrosines in their cytoplasmic tyrosine kinase (TK) domains in a process called signal transduction. This triggers a phosphorylation cascade of the tyrosine residues in multiple downstream molecules and leads to the activation of signal transduction pathways involved in many important cell functions such as proliferation, apoptosis, chemotaxis and adhesion [3]. The presence of *KIT* and *PDGFRA* activating mutations provides the rationale for employing targeted therapies using specific inhibitors (TKI), that can improve recurrence-free survival (RFS) and overall survival (OS) in the majority of patients. The currently used systems for risk stratification are based on tumor size and site, mitotic count and tumor rupture, whereas the prognostic relevance of mutational status is still under debate [4]. CD117 expression occurs in more than 95 % of GISTs bearing *KIT* or *PDGFRA* mutations [5], the remaining 5 % are either CD117 negative or wild-type (WT) for both genes. Thus, to obtain a definite diagnosis additional morphological and/or molecular characterization may be required, such as searching for germline or de novo mutations of *SDH* (succinate dehydrogenase) subunits located on the inner membrane of the mitochondria, or even mutations of the *RAS*-pathway [6]. Among the latter, the frequency of *BRAF* mutations varies from 2 to 13 %, whereas *KRAS* mutations are extremely rare (<0.2 %). Interestingly, concomitant *KRAS* mutations in *KIT*- or *PDGFRA*-mutated GISTs were reported and, based on in vitro experiments, it has been defined that the presence of *RAS* mutations predicts resistance of *KIT*-mutated GISTs to TKI [7]. However, two subsequent analysis in large cohort of GIST patients have not found mutations in *KRAS* codons 12 and 13 or 61 [8, 9]. More recently, one single *KIT/PDGFRA* WT GIST was identified to carry a *KRAS* mutation in codon 12 among 267 patients and associated with an aggressive behavior and resistance to multiple TKI inhibitors [10].

DOG1 (Discovered on GIST-1) is a calcium-dependent chloride channel protein regulating the cholinergic activity of gastrointestinal smooth muscle [11] that is encoded by *ANO1/TMEM16A* on chromosome 11q13; in these tumors its expression shows high sensitivity and specificity [12, 13]. Other functions exerted by *ANO1* include the regulation of both the viability and proliferation of cells overcoming their checkpoints within the cell-cycle [14]. In addition, in DOG1^+^ cells *ANO1* activates alternative signals downstream of the RAS/RAF/MEK/ERK and the insulin-like growth factor (IGF)-dependent pathways [15, 16]. These findings support the hypothesis that DOG1 exerts a definite role in GIST development, regardless of *KIT* and *PDGFRA* activation, whereas its prognostic role is still debated.

Particularly in GISTs lacking CD117 expression and bearing *PDGFRA* mutations [17, 18], DOG1 appears to be a promising tool for diagnosis also of rare variants including gastric spindle and epithelioid-cell *PDGFRA*-mutated GISTs [19]. However, its expression has been little correlated with other risk factors [20–22].

Here we explored the prognostic role of DOG1 in a cohort of patients with GISTs, and evaluated the potential correlation between variable grades of expression and known risk factors for recurrence.

## Methods

### Patients and specimens

Demographic data, histological and immunohistochemical features, as well as mutational status, of 59 patients with GISTs, enrolled at the Medical Oncology Unit of the University of Bari and the IRCCS San Raffaele Pisana in Rome from 2007 to 2014, were collected after obtaining patients’ written informed consent and approval by the Ethics Committee of the University of Bari and the Ethics Committee of IRCCS San Raffaele Pisana in Rome, in accordance with the principles embodied in the Declaration of Helsinki. Selected hematoxylin/eosin stained slides were reviewed to confirm the diagnosis, as well as tumor features including size and histology; CD117 expression was evaluated by immunohistochemistry (IHC). In each sample the number of mitoses was evaluated in 50 consecutive high-power fields (HPFs), while demographic data including tumor staging at diagnosis and follow-up were retrieved from medical records.

### Mutational analysis of *PDGFRA* or *KIT* genes

Tumor specimens were screened for hot-spot mutation sites of *PDGFRA* (exons 12 and 18) and *KIT* (exons 9, 11, 13 and 17) genes. To this end, genomic DNA was isolated from formalin-fixed, paraffin-embedded (FFPE) tissues containing at least 70 % of neoplastic cells. Tumor sections of 8–10 μm were incubated in xylene and then washed with absolute ethanol. DNA was isolated from the air-dried tissues using the QIAamp® DNA FFPE Tissue Kit (QIAGEN, Hilden, Germany) according to the manufacturer’s instructions. Screening of mutations was performed by direct sequencing of the PCR products obtained using primer pairs designed to selectively amplify PDGFRA exons 12 and 18 and KIT exons 9, 11, 13 and 17. PCR reactions were performed using 100 ng of DNA with the primers listed in Additional file 1: Table S1. Mutation analysis was assessed by sequencing of PCR products with the same primers used for PCR reactions and the BigDye® Terminator v1.1 cycle sequencing kit (Applied Biosystems). Sample analysis was performed on an ABI PRISM 310 Genetic Analyzer (Applied Biosystems).

### Immunohistochemistry

The expression of DOG1 was investigated by IHC with the anti-DOG1 monoclonal antibody (MoAb; clone K9, Abcam Cambridge, MA). Five μm FFPE sections of each primary tumor were treated according to the staining Dako Autostainer protocol (Burlington, Ontario, Canada). Briefly, sections were incubated with the anti-DOG1 MoAb at 1:100 dilution for 30 min at room temperature. Stained specimens were analyzed by two pathologists and results were scored according to the Allred scoring system, including a semi-quantitative method to reveal the staining intensity (0 = negative; 1 = weak/trace; 2 = moderate; 3 = strong) and the percentage of positive cells (0 = normal cells; 1 = ≤ 1 %; 2 = 1–10 %; 3 = 11–33 %; 4 = 34–66 %; 5 = 67–100 %). This grading produced a final score [23] that was reported as negative (score 0), weak (score 1–3), moderate (score 4–6) or strong (score 7–8).

### Statistical analysis

Fisher’s exact test was used to evaluate differences between independent groups. The *p*-values for differences between subgroups were adjusted by a permutational test performed in the multitest SAS STAT procedure. Comparison between independent groups was performed by *t*-test, given the Gaussian distribution of data. Statistical analysis was performed using SAS 9.4 software. Recurrence-free survival (RFS) was defined as the time from the date of operation to the date of recurrence and/or distant metastasis. Patients who survived without recurrence and/or metastasis were censored on the date of the last follow-up. RFS was calculated according to the Kaplan-Meier method and the survival distributions were compared by log-rank test. A *p*-value <0.05 was considered statistically significant.

## Results

### Demographics

**Fig. 1:**
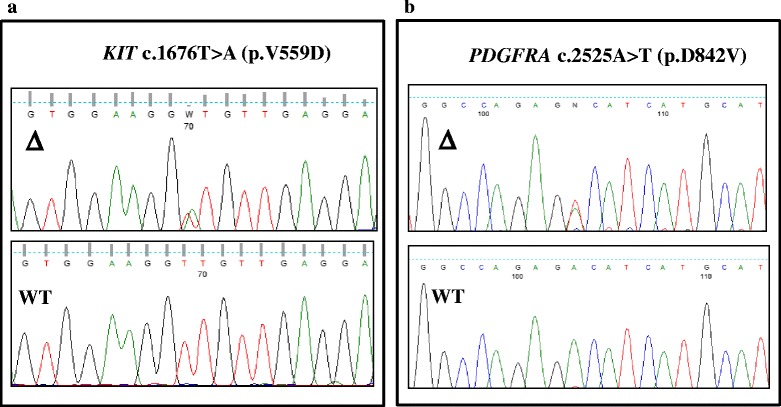
Sequencing analysis. Direct sequencing analysis of the PCR products showing a substitutions of GTT (Val) to GAT (Asp) at codon 559 of KIT gene (*panel* ***a***) and GAC (Asp) to GTC (Val) at codon 842 of PDGFAR gene (*panel* ***b***)

As shown in Table 1, 59 patients with GISTs were enrolled in the study (57 locally-advanced and 2 metastatic; 31 males (52.5 %) and 28 females (47.5 %), median age 63.3 ± 14.6 years). Primary sites included the stomach (*n* = 39; 66.1 %), small (*n* = 12; 20.3 %) and large bowel (*n* = 4; 6.8 %), as well as extra-gastrointestinal sites (*n* = 4; 6.8 %) including the pancreas and retroperitoneum. The histological subtypes included spindle-cell (*n* = 45; 76.3 %), epithelial (*n* = 6; 10.2 %) and mixed (*n* = 8; 13.5 %) variants. Mean tumor size was 8.3 ± 5.5 cm, while the number of mitoses (HPFx50) was ≤5 in 25 (42.4 %), 6–10 in 17 (28.8 %) and ≥10 in 17 (28.8 %) patients. Despite slight variations in CD117 staining intensity, it was considered positive in all patients. Mutational status was available in 53 patients harboring mutations of *KIT* (*n* = 35; 66.1 %) and *PDGFRA* (*n* = 4; 7.5 %) (Fig. 1), whereas in 14 patients (26.4 %) both genes were WT. The identified hot-spot mutations are listed in Additional file 2: Table S2. The average follow-up was 36 + 21 months; 22 % of patients (*n* = 13) had evidence of disease recurrence.

**Table 1:** Clinical pathological features of the 59 GIST cases according to DOG1 expression

Patient characteristics	Cumulative population	DOG1		
		Positive	Negative	
Sex				
Male, n (%)	31 (52.5 %)	19 (48.7 %)	12 (60 %)	*p* = 0.413
Female, n (%)	28 (47.5 %)	20 (51.3 %)	8 (40 %)	
Age (years)				
Mean (±SD)	63.3 (±14.6)	63.1 (±16.9)	63.6 (±12.8)	*p* = 0.8973
Median	67	65	66.5	
Range	28–88	28–88	34–82	
Primary site				*p* = 0.0652
Stomach	39 (66.1 %)	29 (74.3 %)	10 (50 %)	
Small intestine	12 (20.3 %)	8 (20.5 %)	4 (20 %)	
Large bowel	4 (6.8 %)	1 (2.6 %)	3 (15 %)	
Others	4 (6.8 %)	1 (2.6 %)	3 (15 %)	
Tumor size (cm)				*p* = 0.0002
Mean (±SD)	8.3 (±5.5)	10.1 (±5.8)	4.7 (±1.9)	
Median	6	8	4.7	
Range	2–20	3–20	2–10	
Histological subtype				*p* = 0.2
Spindle type	45 (76.3 %)	27 (69.2 %)	18 (90 %)	
Epithelial type	6 (10.2 %)	5 (12.8 %)	1 (5 %)	
Mixed type	8 (13.5 %)	7 (18 %)	1 (5 %)	
Mitoses per 50 HPFs				
≤ 5	25 (42.4 %)	15 (38.5 %)	10 (50 %)	*p* = 0.54
6–10	17 (28.8 %)	13 (33.3 %)	4 (20 %)	
≥ 10	17 (28.8 %)	11 (28.2 %)	6 (30 %)	
Mutated exon				
*KIT* exon 11	30 (56.6 %)	24 (68.6 %)	6 (33.5 %)	*p* = 1
*KIT* exon 9	2 (3.8 %)	2 (5.7 %)	0	
*KIT* exon 13	2 (3.8 %)	2 (5.7 %)	0	
*KIT* exon 17	1 (1.9 %)	0	1 (5.5 %)	
*PDGFRA* exon 12	1 (1.9 %)	0	1 (5.5 %)	*p* = 0.889
*PDGFRA* exon 18	3 (5.6 %)	2 (5.7 %)	1 (5.5 %)	
Wild type	14 (26.4 %)	5 (14.3 %)	9 (50 %)	*p* = 0.009
Not available	6	4	2	

*HPF* high power field of the microscope; *p*-values computed using Fisher exact test or *χ* ^2^ test

### DOG1 expression in GISTs correlates with clinical and pathological features

**Fig. 2:**
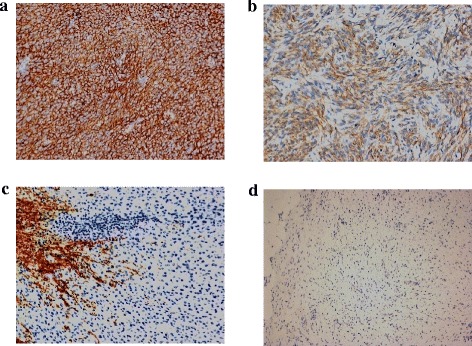
DOG1 measurement according to the Allred scoring system. Representative panels showing the variable DOG1 expression by IHC in patients with GISTs: strong (score: 7–8, panel **a**), moderate (score: 4–6, **b**) and weak (score: 1–3, **c**), while panel **d** shows a DOG1 negative specimen. Magnification is 200x in **a**, **b** and **c**, 100x in **d**

Based on the IHC DOG1 expression, 39 patients (66.1 %) were included in Group A (DOG1^+^) and 20 (33.9 %) in B (DOG1^−^). Representative panels from both groups are included in Fig. 2, showing strong (a), moderate (b) and weak (c) as well as negative (d) cytoplasmic or membranous DOG1 expression. Based on the Allred scoring system, a strong DOG1 expression was demonstrated in 24 Group A patients (Group A1), moderate levels in 12 (Group A2) and a weak expression in 3 patients (Group A3). Levels of DOG1 expression did not correlate with gender, age, primary site, histology, mitoses or mutational status (Table 1). By contrast, tumor size in Group A patients was greater (10.1 ± 5.8 cm) than in Group B (4.7 ± 1.9 cm; *p* = 0.0002), whereas the frequency of the WT status for both *KIT* and *PDGFRA* was lower in Group A than B (14.3 % vs. 50 %; *p* = 0.009).

### DOG1 expression levels and GIST outcome

**Fig. 3:**
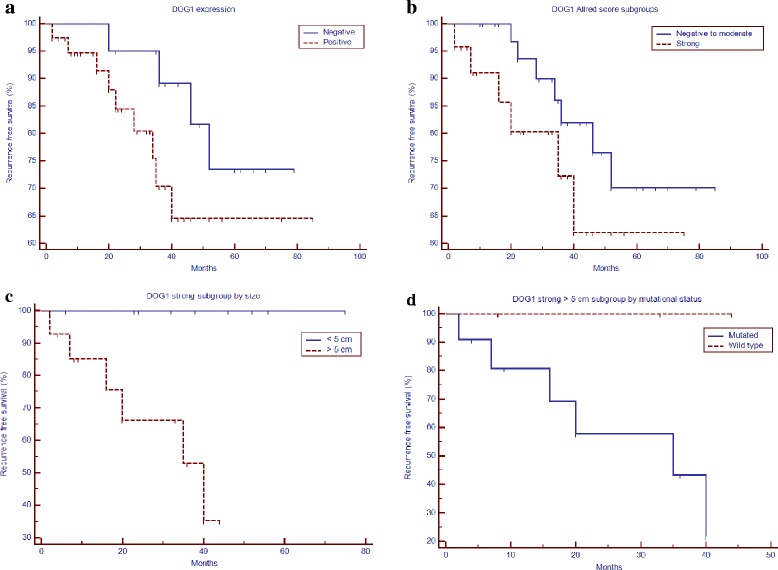
Kaplan-Meier cumulative RFS. **a** The 2-year RFS rate of DOG1-positive patients (Group A) was 84 % (dashed line; *p* = 0.2) as compared to DOG1-negative patients (95 %; solid line; Group B). Disease recurrence occurred in 9/39 and 4/20 patients, respectively. **b** Group A patients were divided by the Allred scoring system into 3 sub-groups (A1, A2 and A3) based on the DOG1 expression levels, indicated as strong, moderate and weak, respectively. The 2-year RFS rate for A1 patients was 80 % (dashed line) compared to 93 % for A2 + A3 + B patients (solid line; *p* = 0.2). Disease recurrence occurred in 6/24 and 7/35 patients, respectively. **c** Group A1 patients were divided by tumor size greater or smaller than 5 cm. The 2-year RFS rate for patients bearing tumors >5 cm was 66 % (dashed line) with 6/14 events compared to 100 % for those with tumors >5 cm (ten patients) (solid line; *p* = 0.01). **d** The 14 Group A1 patients with tumor size > 5 cm were subdivided by mutational status, and the 2-year RFS rate for those (*n* = 11) harboring mutations was 58 % (solid line) compared to 100 % for the 3 WT patients (dashed line, *p* = 0.16). Recurrence occurred in 6/11 Group A1 patients

DOG1 expression was investigated in relation to a potential predictive role with respect to the onset of recurrence. Nine Group A patients (23 %) and four Group B (20 %) recurred during follow-up, yielding 2-year RFS rates of 84 and 95 % respectively. The cumulative RFS curve in Group A patients was worse, although not significantly so, compared to Group B (Fig. 3a). We also investigated the relationship between DOG1 levels and RFS, and found that Group A1 patients had the worst 2-year (panel b) RFS rate (80 %; 6/24) as compared to the other groups (93 %; 7/35). Further analyses were performed to investigate whether the previously described correlation of DOG1 expression with both tumor size and mutational status was associated with the RFS. Therefore, Group A1 patients were subdivided by tumor size greater (*n* = 14) or smaller (*n* = 10) than 5 cm. As shown in panel c, the Kaplan-Meier survival curve revealed 2-year RFS rates of 66 % (6/14 events) and 100 % respectively (*p* = 0.01). Moreover, among A1 patients with a tumor size >5 cm (panel d), those carrying a *KIT* or *PDGFRA* mutation (*n* = 11) had a worse prognosis than the WT (*n* = 3), the 2-year RFS rates being 58 and 100 % respectively. The trend to statistical significance (*p* = 0.16) was, however, influenced by the sample size.

## Discussion

GISTs are rare tumors with morphological, histological and molecular features that strongly influence both the outcome and risk of recurrence. Since the discovery of the role of oncogenic mutations of *KIT* and *PDGFRA*, targeted therapy with TKI has significantly increased the OS in the majority of patients. However, WT GISTs or those harboring rare mutations often experience progression or recurrence and so a better risk stratification is needed in order to plan adequate therapeutic strategies.

Measurement of DOG1 expression by IHC has been associated with a higher diagnostic sensitivity and specificity than CD117, allowing the diagnosis of GISTs in about 30 % of CD117-negative patients [18]. Its expression has been described in both normal and malignant tissues, although its prognostic role is still being debated. The DOG1 protein mediates the receptor-activated chloride current whose levels modulate the cell proliferation by affecting the retinoblastoma (Rb) tumor suppressor protein phosphorylation [14, 24, 25], or by activating the MEK/ERK pathway [15]. In addition, xenograft DOG1^−/−^ models of GISTs show an impaired cell proliferation as a consequence of the decreased IGF binding protein-5 levels [16], that inhibit IGF-mediated downstream signals by trapping both IGF1 and IGF2 [26]. These findings suggest that DOG1 over-expression provides a proliferative advantage to malignant stromal cells, and increased levels could negatively influence prognosis.

Here, we describe results from an observational study based on evaluation of the clinical, pathological and molecular features of 59 GIST patients and any correlations with DOG1 expression. Approximately 66 % of CD117^+^ samples showed a strong DOG1 expression, in agreement with previous studies describing its variable accumulation in 60–99 % malignant cells. The reported variability in DOG1 expression was mostly attributed to different monoclonal antibodies used for IHC analyses, as well as to the intrinsic characteristics of the specimens [10, 21, 27]. In accordance with previous studies [19–21, 28], our data showed that DOG1 expression is unrelated to gender, age, primary site, histological subtypes and mitoses, although a significant correlation was demonstrated with large tumors harboring an unfavorable mutational status. Tumor size is already considered a prognostic factor for the definition of high-risk disease [29–31]. However, the prognostic role of the mutational status is still under debate and not included in the current risk stratification systems. It is noteworthy that the presence of the homozygous *KIT* exon-11 mutation predicts an aggressive disease course, in particular when deletions affect both codons 557–558 [32]. By contrast, the majority of *PDGFRA* mutated GISTs show a benign course [33]. Our data support those recently published in a meta-analysis on 1487 patients [34], proving that GISTs bearing *KIT* mutations have a significantly poorer prognosis than either *PDGFRA* mutated or WT GISTs. Moreover, Rìos-Moreno et al. reported that the WT genotype was prevalent in DOG1^−^/CD117^−^ patients [35]. We demonstrated a more favorable post-operative 2-year RFS rate in DOG1-negative patients than DOG1-positive patients (*p* = 0.02). These findings were in line with previous results [36] that reported a significant association between DOG1 expression and high-risk tumors. We stratified DOG1 positive patients in relation to the Allred scoring system to identify those with a higher risk of recurrence; in our study patients with a strong DOG1 expression, tumor size ≥ 5 cm and mutations of *KIT* or *PDGFRA* had a worse prognosis.

The genetic landscape of GIST patients should be further investigated. In particular, given the correlation between DOG1 expression and the activation of the downstream RAS/RAF/MEK/ERK signaling pathway, the clinical significance of activating *RAS* mutations remains to be better elucidated for its therapeutic relevance, as already widely investigated in other tumors [37].

## Conclusions

In conclusion, in our patients a high DOG1 expression correlated with an aggressive malignant phenotype of GISTs. Thus, measurement of DOG1 expression would be helpful in clinical practice to predict the recurrence risk in GIST patients. We believe that the Allred scoring system could be integrated in current risk stratification systems to achieve a better identification of patients at increased risk of recurrence.

### Availability of data and materials

The datasets supporting the conclusions of this article are included within the article and its additional files.

## Additional files

Additional file 1: Table S1. Polymerase Chain Reaction primers, product size and reaction conditions for amplification and direct sequencing for assay of *KIT* and *PDGFRA* genes. (DOC 38 kb) Additional file 2: Table S2. Genotype of GIST patients carrying mutations. (DOCX 19 kb)

## Abbreviations

ANO1: anoctamin-1
DOG1: discovered on GIST-1
FFPE: formalin-fixed, paraffin-embedded
GISTs: gastrointestinal stromal tumor
HPFs: high-power fields
IGF: insulin-like growth factor
IHC: immunohistochemistry
KIT: v-kit Hardy-Zuckerman 4 feline sarcoma viral oncogene homolog
OS: overall survival
PCR: polymerase chain reaction
PDGFRA: platelet-derived growth factor receptor, alpha polypeptide
Rb: retinoblastoma
RFS: recurrence-free survival
SDH: succinate dehydrogenase
TKI: tyrosine kinase inhibitors
TMEM16A: transmembrane member 16A
WT: wild-type
