# Supplementary material for: Parallelism of DOG1 expression with recurrence risk in gastrointestinal stromal tumors bearing KIT or PDGFRA mutations
Source: BMC Cancer. 2016 Feb 11;16:87. doi: 10.1186/s12885-016-2111-x (PMC4750215; doi:10.1186/s12885-016-2111-x)
Supplement: Additional file 1: Table S1. — Polymerase Chain Reaction primers, product size and reaction conditions for amplification and direct sequencing for assay of KIT and PDGFRA genes. (DOC 38 kb) [file 12885_2016_2111_MOESM1_ESM.doc]

**Table S1.** Polimerase Chain Reaction primers, product size and reaction conditions for amplification and direct sequencing for assay of *KIT* and *PDGFRA* genes.

| **Gene** | **Exon** | **Primer Sequence** | **Product size (bp)** | **PCR annealing temp (C˚)** |
| --- | --- | --- | --- | --- |
| ***KIT*** |  |  |  |  |
|  | 9 | F 5' ttcctagagtaagccaggg 3' | 299 | 53 |
|  |  | R 5' aatcatgactgatatggt 3' |  |  |
|  | 11 | F 5' caggtaaccatttatttgt 3' | 262 | 53 |
|  |  | R 5' tacccaaaaaggtgacatgg 3' |  |  |
|  | 13 | F 5' cttgacatcagtttgccag 3' | 256 | 53 |
|  |  | R 5' tttataatctagcattgcc 3' |  |  |
|  | 17 | F 5' gttttcactctttacaagt 3' | 223 | 53 |
|  |  | R 5' cctttgcaggactgtcaagc 3' |  |  |
|  |  | |  |  |
| ***PDGFRA*** | 12 | F 5' tccagtcactgtgctgcttc 3' | 261 | 54 |
|  |  | R 5' gcaagggaaaagggagtctt 3' |  |  |
|  | 18 | F 5' tcagctacagatggcttgatc 3' | 215 | 55 |
|  |  | R 5' tgaaggaggatgagcctgacc 3' |  |  |
