# Supplementary material for: Parallelism of DOG1 expression with recurrence risk in gastrointestinal stromal tumors bearing KIT or PDGFRA mutations
Source: BMC Cancer. 2016 Feb 11;16:87. doi: 10.1186/s12885-016-2111-x (PMC4750215; doi:10.1186/s12885-016-2111-x)
Supplement: Additional file 2: Table S2. — Genotype of GIST patients carrying mutations. (DOCX 19 kb) [file 12885_2016_2111_MOESM2_ESM.docx]

**Table S2.** Genotype of GIST patients carrying mutations.

| **Gene** | **Exon** | **Patient** | **Nucleotide change** | **Aminoacid change** |
| --- | --- | --- | --- | --- |
| ***KIT*** | *9* | 1 | c.1441A>G | p.S481G |
|  |  | 2 | c.1509_1510insGCCTAT. | p.Y503_F504insAY |
|  | *11* | 3 | c.1672_1716del45 | p.K558_D572del |
|  |  | 4 | c.1648_1674del27 | p.K550_K558del |
|  |  | 5 | c.1665_1676del12 | p.Q556_V559del |
|  |  | 6 | c.1667_1672delAGTGGA | p.W557_K558del |
|  |  | 7 | c.1652_1669del18 | p.P551_W557>R |
|  |  | 8 | c.1657_1716del59 | p.Y553_D572del |
|  |  | 9 | c.1652_1654delCCA | p.P551_M552>L |
|  |  | 10 | c.1679_1681delTTG | p.V560del |
|  |  | 11 | c.1756_1757ins36 | p.P585_R586ins12 |
|  |  | 12 | c.1661_1675del15 | p.E554_K558del |
|  |  | 13 | c.1676T>C | p.V559A |
|  |  | 14 | c.1748_1749ins21 | p.E583_F584insPYDHKWE |
|  |  | 15 | c.1703A>G | p.Y568C |
|  |  | 16 | c.1660_1674del15 | p.E554_K558del |
|  |  | 17 | c.1727T>C | p.L576P |
|  |  | 18 | c.1755_1756ins42 p | P585_R586ins14 |
|  |  | 19 | c.1727T>C | p.L576P |
|  |  | 20 | c.1727T>C | p.L576P |
|  |  | 21 | c.1679T>A | p.V560D |
|  |  | 22 | c.1669_1674delTGGAAG | p.W557_K558del |
|  |  | 23 | c.1661_1675del15 | p.E554_K558del |
|  |  | 24 | c.1740_1741ins24 | p.H580_K581insPTQLPYDH |
|  |  | 25 | IVS10-3_1672del28 | aberrant splicing |
|  |  | 26 | c.1652_1663del12 | p.P551_V555>L |
|  |  | 27 | c.1648_1677del30 | p.K550_V559del |
|  |  | 28 | c.1676T>A | p.V559D |
|  |  | 29 | c.1676T>A | p.V559D |
|  |  | 30 | c.1648_1671del24 | p.K550_W557del |
|  |  | 31 | c.1654_1659delATGTAT | p.M552_Y553del |
|  |  | 32 | c.1669T>G | p.W557G |
|  | *13* | 33 | c.1924A>G | p.K642E |
|  |  | 34 | c.1924A>G | p.K642E |
|  | *17* | 35 | c.2466T>A | p.N822K |
| ***PDGFRA*** | *12* | 36 | c.1759G>A | p.E587K |
|  | *18* | 37 | c.2525A>T | p.D842V |
|  |  | 38 | c.2525A>T | p.D842V |
|  |  | 39 | c.2525A>T | p.D842V |
